# It Takes More Than One Swallow to Make a Summer: Measures to Foster Girls' and Women's Pathways Into STEM

**DOI:** 10.3389/fpsyg.2019.01844

**Published:** 2019-08-16

**Authors:** Silke Luttenberger, Petra Steinlechner, Bernhard Ertl, Manuela Paechter

**Affiliations:** ^1^Institute in Early Childhood and Primary Teacher Education, University College of Teacher Education Styria, Graz, Austria; ^2^Educational Psychology, Institute of Psychology, University of Graz, Graz, Austria; ^3^Learning and Teaching with Media, Department of Education, Universität der Bundeswehr München, Neubiberg, Germany

**Keywords:** gender, STEM, self-efficacy, interest, career choice, career counseling, STEM teaching

## Theory Ties Research and Practical Interventions Together

For decades, the proportion of women in STEM professions (Science, Technology, Engineering, Mathematics) has remained at approximately one fourth in the European Union – an alarmingly low number (Center of Excellence Women and Science, 2014). With labor markets continuing to communicate an increasing need in STEM workforces, this low number signals unfulfilled talent that is otherwise greatly needed in many critical fields. Effective interventions are needed (Walsh and Heppner, 2006) to foster girls' and women's pathways into STEM. Yet, when it comes to the implementation of interventions and their effectiveness, current efforts leave a lot to be desired. The present article describes how girls and women can be encouraged to consider STEM professions as real options.

**Figure 1:**
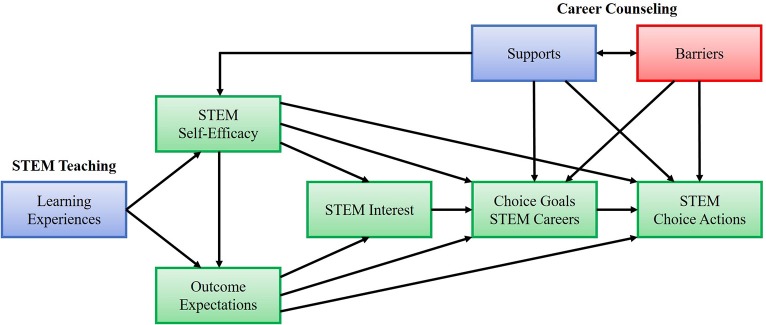
Social Cognitive Career Theory: Interest and Choice Model.

There are many situations where women lose interest or fail to build up interest in STEM over their formative years from early childhood to school and tertiary education. The Social Cognitive Career Theory (Lent et al., 1994; Lent and Brown, 2019) stresses key variables for the development and realization of career interest and goals (Figure 1). Important personal factors are STEM self-efficacy and outcome expectations for entering a STEM career. These factors are related to STEM interest, which in turn may lead to STEM career goals. “Building self-efficacy for math and science and fostering positive and realistic outcome expectations would lead to realistic and investigative interests, that would, in turn, lead to STEM career goals and preparation for, and entry into, a STEM occupation” (Fouad and Santana, 2017, p. 27).

However, pathways into STEM careers are not only related to personal factors. Structural or social factors may work as barriers and filter out girls and women from STEM careers (Watt et al., 2006; Turner et al., 2019).

The present article investigates closer how girls and women can be supported in the formation of interest in a field, to a certain career goal, to a specific choice of action.

## STEM Pathways: From Interest to a Career Goal and Choice of Action

### Interest

Positive STEM experiences in school are a key to the development of interest and career goals in STEM (Fouad et al., 2010; Ertl et al., 2017; Luttenberger et al., 2019). Ideally, they should raise interest as well as self-efficacy in STEM (compare Figure 1).

Intervention studies concerning STEM teaching found positive effects on students' interest and motivation with measures such as inquiry-based STEM teaching (UNESCO, 2017), improving teaching using STEM pedagogy (Gaspard et al., 2015), bringing real-life applications into the classroom (Taskinen et al., 2013), hands-on activities (Lee and Erdogan, 2007), or design-based learning with laboratory and workshop experiences (Vongkulluksn et al., 2018). Furthermore, informal learning environments such as STEM summer camps were an effective means to spark middle-school students' interest in STEM (Mohr-Schroeder et al., 2014). In a meta-analysis by Furtak et al. (2012), inquiry-based STEM teaching was found to be highly effective for learning and the development of interest.

It seems to be important that interventions to foster interest and raise career aspirations start early, i.e., in primary education (even though to date, programs have been mainly implemented starting in middle school). Primary school students were the focus of two intervention studies: Here, design-based learning via Makerspaces (Vongkulluksn et al., 2018) was able to spark students' interest in STEM activities. However, even though self-efficacy and interest remained moderately high, they declined over the Makerspace intervention semester. One of the few studies which combined indirect (learning experiences and STEM) and direct interventions (career counseling) was carried out by Panayiotou and Eteokleous-Grigorio (2017) with a robotic course as didactic intervention. Although it found increases in STEM interest, positive attitudes, and motivation, no effects on career aspirations were identified.

Altogether, STEM teaching has to be designed carefully to raise not only the interest in STEM, but also in STEM careers (Fouad et al., 2010). Of note is the general lack of intervention studies which combine STEM teaching and career counseling.

### Career Goals

Career counseling is assumed to have a direct impact on students' career choices because students will only strive for professions they are aware of (Herr et al., 2004).

Students generally require more information about STEM professions, and have to actively search for job-related information. In an intervention study by Turner and Lapan (2002), a computerized training for middle school students was developed to foster interest and self-efficacy in STEM professions for females. A strength of this study lies in its discussion of the training results for career counseling. It was possible to identify girls who are in fact interested in a STEM career but lack social support. Yet, only short-term gains were measured after one week in this study; its long-term effects remain unclear. Painter et al. (2006) focused on raising students' interest in scientific careers in STEM. They found positive effects of contextualized science materials and interactions with scientists among 7th and 10th graders (about 10% reported having previously interacted with scientists at school).

All in all, career counseling is essential for informing students about career choices. It should aim at the development of realistic expectations about STEM professions that match individual interest.

### Specific Choice of Action

Teachers play a crucial role in supporting students on the pathway from career goals to choice of actions. Career teachers' lack of knowledge often prevents the choice of STEM professions (Cleaves, 2005). The higher teachers' encouragement and help when needed, the higher the motivation of girls and women to explore STEM careers (Blustein et al., 2013). In general, girls and women experience less support to develop and pursue STEM-related career goals. Also parental beliefs and stereotypes in particular can support or hinder career choices in STEM (Ertl et al., 2017).

Social Cognitive Career Theory (Lent et al., 1994) points at the importance of role models. A lack of female role models (family members, peers etc.) can decrease the sense of belonging in STEM (Blickenstaff, 2005). In an intervention study by Robnett et al. (2018) instrumental and socio-emotional mentoring were able to foster women's sense of belonging to a STEM community.

## Starting Early: The Importance of Early Career-Related Learning Experiences for STEM Pathways

Most interventions focus on students in upper secondary education from age fourteen onwards (DeWitt and Archer, 2015), even though career choices are unlikely to change dramatically by this age. Career aspirations mostly have already formed by the age of 13. After this, it is increasingly difficult to interest students in STEM (Lindahl, 2007). Therefore, the critical age period during which aspirations are formed is during primary and lower secondary education (Lindahl, 2007; DeWitt and Archer, 2015).

In the first phase of career orientation, interest in both STEM learning and STEM aspirations are still unstable (Ardies et al., 2015). Interest in STEM typically shows a downward trend from primary school on (Taskinen et al., 2013). Interventions in primary education focus mainly on STEM teaching and learning (Panayiotou and Eteokleous-Grigorio, 2017; Vongkulluksn et al., 2018) or informal learning experiences by using authentic STEM workplaces (Roberts et al., 2018). They show that learning experiences are needed at an early age to support the transition from career interest to choice of goals. Girls who aspire to a STEM career as early as primary school are more likely to choose a STEM profession (Schoon, 2001). However, the relationship between interest and career aspirations has seldom been the focus of interventions. This is why there is a need for studies providing advice on how to foster not only interest in STEM, but STEM career aspirations as well (Panayiotou and Eteokleous-Grigorio, 2017).

A problem with many intervention studies is that they often appeal only to those students who are already interested in STEM, and not to those who are skeptical about these fields. Interventions should aim at all girls and students, at interested students, as well as not-so-interested ones. They should start at an early age, aim to raise and sustain interest, and transform it into career goals and choices of action. Real-life experiences with STEM, e.g., hands-on experiences, apprenticeships, career counseling, and role models can expand girls' knowledge about STEM and professions while maintaining effective levels of interest (UNESCO, 2017).

## Focusing not Only on Personal Factors but Also on Removing External Barriers

Social Cognitive Career Theory provides an empirical basis for interventions to foster STEM interest and goals (Lent et al., 2018). Interventions and attempts in education to foster the proportion of women in STEM mostly focus on personal factors, e.g., self-efficacy, outcome expectations, interest, or STEM belonging. Social Cognitive Career Theory also stresses the importance of social factors. Turner and Lapan (2002) showed that parents may either support their daughters on a pathway into STEM or put barriers along it. In the same way, not only parents, but other family members, peers, teachers as well as future employers may offer support or create barriers. The identification of barriers and support is important for transferring interest into choices of action and promoting females' participation in STEM (Fouad et al., 2010). Contextual and social factors play different valuable roles, indirectly and directly, in fostering women's STEM interest and goals (Lent et al., 2018).

## Conclusion

There are a multitude of factors that influence the career paths of girls and women. Fostering girls' pathways into STEM requires continuous and multiple interventions that start at an early age and address personal as well as social factors. As Social Cognitive Career Theory points out, they should take into account key variables in the development and realization of career wishes, the formation of interest in a field, and the formation of career goals to coincide with specific choices of action.

## Author Contributions

SL, PS, BE, and MP have made a substantial, direct and intellectual contribution to the work, and approved it for publication.

### Conflict of Interest Statement

The authors declare that the research was conducted in the absence of any commercial or financial relationships that could be construed as a potential conflict of interest.
